# Evaluation of the prehospital diagnostic accuracy of a novel point-of-care test for NT-proBNP, D-dimer and H-FABP and large-vessel occlusion risk assessment (LVOCheck-EVA): a protocol for a multicenter prospective observational study in patients suspected of having a stroke

**DOI:** 10.3389/fneur.2025.1697711

**Published:** 2025-12-08

**Authors:** Kian Röhrs, Maximilian Kaffes, Fulvio Bondi, Martina Zuber, Dorothee Kübler-Weller, Lisa Haacke, Robert Röhle, Erin Dirk Sprünken, Eugen Schwabauer, Matthias Wendt, Carmen de Jesus Gil, Jean-Charles Sanchez, Joan Montaner, Heinrich J. Audebert, Joachim E. Weber

**Affiliations:** 1 Department of Neurology, Charité—Universitätsmedizin Berlin, Corporate Member of Freie Universität Berlin and Humboldt-Universität zu Berlin, Berlin, Germany; 2 Center for Stroke Research Berlin, Charité—Universitätsmedizin Berlin, Corporate Member of Freie Universität Berlin and Humboldt-Universität zu Berlin, Berlin, Germany; 3 Institute of Biometry and Clinical Epidemiology, Charité—Universitätsmedizin Berlin, Corporate Member of Freie Universität Berlin and Humboldt-Universität zu Berlin, Berlin, Germany; 4 Department of Neurology, Vivantes Klinikum Neukölln, Berlin, Germany; 5 Department of Neurology, Unfallkrankenhaus Berlin, Berlin, Germany; 6 Advanced Brain Companion Diagnostics S.L. (ABCDx), Project Partner, Barcelona, Spain; 7 Department of Medicine, Faculty of Medicine, University of Geneva, Geneva, Switzerland; 8 Institute de Biomedicine of Seville, IBiS/Hospital Universitario Virgen del Rocío/CSIC/University of Seville, Seville, Spain; 9 Department of Neurology, Hospital Universitario Virgen Macarena, Seville, Spain; 10 Berlin Institute of Health at Charité—Universitätsmedizin Berlin, Berlin, Germany; 11 German Center for Cardiovascular Research (DZHK), Partner Site Berlin, Berlin, Germany

**Keywords:** mobile stroke unit, biomarkers, stroke, cerebrovascular disease, large vessel occlusion, emergency medicine, point-of-care test, golden hour

## Abstract

**Introduction:**

Acute ischemic stroke (AIS) is a leading cause of permanent disability in adults and one of the most time-sensitive emergencies in modern medicine. Rapid diagnosis and initiation of thrombolytic therapy, as well as immediate access to mechanical thrombectomy for patients with large-vessel occlusion (LVO), are critical determinants of favorable outcomes. While Mobile Stroke Units (MSUs) – ambulances equipped with computed tomography (CT) imaging – have demonstrated efficacy in improving outcomes, their deployment is often constrained to urban environments due to cost-efficiency considerations. Blood biomarkers offer a potentially cost-effective alternative for stroke diagnosis and subtyping. Especially for patients with LVO, a prehospital biomarker-based identification could enable a streamlined transport strategy directly to thrombectomy-capable stroke centers. In this study, we evaluate the diagnostic accuracy and feasibility of a novel point-of-care test (POCT) in predicting LVO stroke, along with the levels of vascular biomarkers—NT-proBNP, D-Dimer, and H-FABP—from ultra-early whole-blood samples of patients with suspected stroke in a prehospital setting. The test integrates a blood-based multiplex lateral flow assay (LFA) with clinical decision support (CDS) software, accessible through a Mobile Application (App). The study was registered in the German Clinical Trials Register (DRKS-ID: DRKS00037840).

**Methods and analysis:**

This multicenter prospective observational study will include 800 patients with suspected stroke, enrolled within 24 h after symptom onset. Participants will be recruited at three Mobile Stroke Unit (MSU) sites in Berlin, Germany. Prehospital blood samples will be analyzed directly in the ambulance using the LVOCheck device. The test achieves its performance by quantifying the blood levels of the vascular biomarkers (NT-proBNP, D-Dimer, and H-FABP), and inputting these concentrations, along with neurological assessment score and patient-specific medical information such as age and blood pressure, to output predictive information on the probability of LVO stroke, together with all the input data. Additional clinical data, including final diagnoses, will be documented in electronic case report forms (eCRFs). The diagnostic performance of LVOCheck will be evaluated through comprehensive statistical analysis of the combined biomarker and clinical data, including assessment of sensitivity, specificity, and predictive models.

**Discussion:**

This real-world study aims to evaluate the diagnostic accuracy and feasibility (including human factors and usability) of LVOCheck, a portable, non-invasive multiplex POCT at prehospital settings, such as an ambulance, in the prediction of acute LVO diagnosis and triage of suspected acute LVO stroke patients. Utilizing Mobile Stroke Units (MSUs) as recruiting sites enables the analysis of ultra-early biomarker levels from prehospital whole-blood samples combined with patient-specific medical information to provide a predictive risk of LVO stroke. A cost-effective and practical POCT could provide additional biochemical information to support prehospital LVO identification in the future, thereby potentially assisting emergency medical services in transport decisions regarding direct transfer to thrombectomy-capable centers. This may improve triage efficiency, treatment metrics and outcomes in both urban and rural settings.

**Clinical trial registration:**

https://www.drks.de/search/de/trial/DRKS00037840/details, Identifier DRKS00037840.

## Introduction

Neurological diseases, and particularly cerebrovascular conditions like acute ischemic stroke (AIS) are major contributors to global disease burden (1). Due to the low tolerance to hypoxia of brain tissue, stroke is one of the most time-sensitive emergencies in modern medicine. To limit tissue injury, reduce disability, and decrease mortality, effective revascularization therapies like intravenous thrombolysis (IVT) and, in cases of large vessel occlusion (LVO), mechanical thrombectomy (MT) must be administered within established therapeutic time windows after symptoms onset.

Given the time-critical nature of these evidence-based treatments for AIS, they must be initiated at the earliest possible moment following symptom onset and transportation and diagnostic delays must be minimized (2–4). The treatment efficacy of revascularizing therapies is especially impactful, when administered during the critical first hour after onset, known as “golden hour of stroke” (5, 6). The first point of contact for stroke patients after symptom onset typically is the emergency medical team, which may include paramedics and in some countries prehospital emergency physicians depending on the local healthcare system. Despite early first contact with the healthcare system in many cases, treatment is often delayed due to the need for transportation to a stroke-capable hospital and, in cases of LVO, often a secondary transfer to a thrombectomy-capable center. Starting reperfusion therapy later after onset is associated with reduced intervention effectiveness in many cases. This is particularly evident in the near-exponential decline in IVT efficacy observed within the “golden hour” (7). Notably, recent clinical trials have shown how medium and distal-vessel occlusion may not benefit from MT as much as strict LVO cases do, which support the demand of improved screening LVO cases (8, 9).

Mobile Stroke Units (MSUs) are specialized ambulances equipped with computed tomography (CT) scanners, angiography functionality, and point-of-care (POC) testing to allow for immediate stroke diagnosis and prehospital therapy initiation. The use of MSUs reduces alarm-to-treatment times, increases overall intervention rate but particularly within the first hour of symptom onset (“golden hour”), and improves patients outcomes compared to conventional emergency care (5, 10–12). Since 2011, the Berlin Fire Department has implemented three MSUs (Stroke Emergency Mobiles, STEMO), providing a successful model for prehospital stroke management in a metropolitan area (13).

For LVO, MT is the guideline-recommended treatment alongside IVT (3, 4). While IVT is widely available at regional stroke units, the availability of MT is comparatively limited and is restricted to thrombectomy-capable stroke centers.

Current evidence suggests that neither grounding transportation strategies to primary stroke centers (PSC, “Drip-and-Ship”) nor thrombectomy-capable stroke centers (TCSC, “Mothership”) for severely affected patients with suspected LVO, demonstrate significant outcome improvements for either of the groups at a population level (14). However, reliable and early LVO detection outside the hospital setting could improve outcomes by allowing direct transport to TCSCs for select patients. In current practice—particularly in settings without mobile stroke units (MSUs)—prehospital triage for large vessel occlusion (LVO) mainly relies on clinical severity-based tools such as the RACE or LAMS scales. However, these clinical scales have limited diagnostic accuracy for detecting LVO, often leading to both under- and over-triage. As highlighted by Helwig et al. (15), MSU-based imaging triage substantially outperforms such clinical assessments in identifying LVOs, but MSUs currently remain to be economically feasible only in metropolitan regions (16, 17). Thus, reliable and early LVO detection outside the hospital setting remains an unmet need, particularly in non-metropolitan areas. Blood-based biomarkers represent a promising alternative, potentially offering minimally invasive, “easy-to-use,” and economical options for stroke and LVO identification. However, the sensitivity of individual biomarkers is often insufficient for clinical use. Combining multiple biomarkers might enhance the diagnostic accuracy of blood-based tests for stroke and its subtypes. This study focuses on assessing the diagnostic potential of three previously reported biomarkers to enhance the detection of LVO.

N-terminal pro-B-type Natriuretic Peptide (NT-proBNP) is a cleavage product of proBNP, which is primarily expressed by cardiomyocytes and is released into the bloodstream in response to increased myocardial wall stress, as observed in conditions like heart failure. Elevated levels of NT-proBNP are frequently observed in cardioembolic strokes, which often present with LVO (18–20).

D-Dimer is a fibrin degradation product generated during fibrinolysis. It reflects active clot formation and breakdown. Elevated D-Dimer levels have been associated with acute ischemic stroke, particularly in cardioembolic subtypes, and may furthermore correlate with stroke severity and prognosis (21, 22). However, D-Dimer elevation lacks specificity, as levels can be elevated in various conditions (pulmonary embolism, thrombosis, trauma, infection).

Heart-type fatty acid-binding protein (H-FABP) is rapidly released following neurological injury and may help distinguish ischemic stroke from so-called stroke mimics (23). Stroke mimics are a heterogeneous group of diseases (e.g., migraine or seizures), that can present with stroke-like symptoms without evidence of a permanently impaired brain perfusion.

We hypothesize, that the combined assessment of H-FABP, D-Dimer and NT-proBNP with clinical parameters in patients with suspected LVO-stroke can improve prehospital LVO identification.

Historically, stroke biomarker research has focused primarily on measurements of blood samples obtained after hospital admission (24). As a result, insights into the early and ultra-early dynamics of blood-based biomarkers are limited. This limitation may have contributed to the lack of translational success in the field of biomarker research in stroke despite several decades of research. The advent of MSUs now allows to address this limitation.

Studies on the integration of simpler and widely accessible platforms, such as smartphones, for image capture and biomarker analysis, showed promising results with respect to a fast and accurate stroke diagnosis in ambulances and other prehospital environments (25). The LVOCheck technology utilizes an integrative design, which combines a blood-based multi-biomarker single LFA test, with a Mobile Application (App). This integration might provide enhanced predictive capabilities in pre-hospital stroke care, facilitating early and accurate prediction of LVO strokes.

In this study, we aim to evaluate the diagnostic accuracy and feasibility of a novel POCT for predicting LVO stroke within existing stroke care protocols aboard MSUs. The tool integrates clinical data collected by emergency responders with measurements of three vascular biomarkers—NT-proBNP, D-Dimer, and H-FABP—to generate a combined probability score, assisting healthcare providers in pre-hospital prediction and triage of suspected acute LVO stroke patients. This approach facilitates early biomarker assessment directly at the POC, simulating the intended future use case in standard ambulances.

## Methods and analysis

### Aim of the study

The primary aim of this study is to evaluate whether the prehospital, field-based, multiplex POC LVOCheck device, which quantifies the blood levels of three vascular biomarkers—NT-proBNP, D-Dimer, and H-FABP—can effectively assess the risk of an LVO stroke in patients with suspected acute stroke. These biomarker levels, combined with neurological assessment scores and other patient-specific medical information (age and blood pressure), are inputted into a Mobile App to generate a risk estimate. Based on preliminary data, we hypothesize that this approach can achieve a specificity above 92.5% and a sensitivity above 50% for LVO prediction.

The hypothesis will be tested by assessing biomarker levels during the ultra-early prehospital phase in patients suspected of acute stroke, as based on the initial dispatch center telephone triage, regardless of the final diagnosis. Considering that many prior studies have focused on measuring biomarker levels in blood samples collected during later stages of AIS, primarily after hospital admission, the levels of biomarkers in the early prehospital phase of stroke remain largely unexplored.

The study methodology builds upon data from prior investigations, BIO-FAST (26) which was presented at ESOC 2021, as well as the preceding pilot study LVOCheck-Opti (27), which is currently under evaluation. These studies informed the development of lateral flow assays with clinically relevant detection thresholds for the three biomarkers under investigation.

The LVOCheck-Eva study will assess the diagnostic accuracy of the developed POCT tool in real-world conditions. This evaluation will help determine the feasibility (including human factors and usability) of integrating biomarker-based LVO prediction into standard stroke operating procedures for emergency medical services.

Given the rapid progression of neuronal injury after symptom onset, the sensitivity and specificity of the test are expected to improve with increasing time from symptom onset. It is furthermore hypothesized that most false-positive results will occur in non-LVO AIS patients rather than in those with hemorrhagic stroke or stroke mimics.

## Hypotheses

### Primary study hypothesis

We hypothesize that combining POCT-based measurements of NT-proBNP, D-Dimer, and H-FABP with patient-specific clinical data, will enable pre-hospital identification of LVO in suspected stroke patients with a sensitivity of 50% and a specificity of 92.5%.

### Secondary study hypotheses

The POCT biomarker measurements of NT-proBNP, D-Dimer, and H-FABP in whole blood are equivalent to the values obtained in fresh plasma analysis.

Incorporating LVOCheck into prehospital standard of care (SOC) can support triage decision-making, and be effectively integrated into routine clinical workflows, as assessed through qualitative assessment of HCPs feedback.

## Study design

LVOCheck-Eva is a multicenter prospective observational study enrolling patients with suspected stroke within 24 h of symptom onset, identified through initial telephone-based dispatch center evaluation. The study aims to evaluate the sensitivity and specificity of a newly developed multiplex POCT that integrates NT-proBNP, D-Dimer and H-FABP measurements from whole-blood and plasma samples with patient-specific medical data for LVO risk prediction, in a real-world prehospital setting.

## Study population

### Patient recruitment

The study intends to recruit 800 patients suspected of suffering from acute stroke, identified through dispatch center evaluations, between October 2025 and November 2026, at three mobile stroke unit locations in Berlin. These MSUs are staffed and equipped to provide comprehensive prehospital stroke diagnosis and therapy. Initial triage is conducted by the dispatch center of the Berlin Fire Department using a standardized stroke symptom checklist during telephone assessment.

### Inclusion criteria

Participants qualify for inclusion in the study if they meet all the following criteria:

- Age ≥ 18 years.
- Suspected stroke based on the initial fire department dispatch center evaluation.
- Symptom onset or last known well (if onset time is uncertain) ≤ 24 h.
- A vascular access has been established as part of routine clinical care.
- Blood sample collection possible prior to treatment initiation at the ambulance.

### Exclusion criteria

Participants are excluded from the study if they meet any of the following criteria:

- Refusal to participate or withdrawal of written consent, either personally or through a legal representative.
- Blood sampling is not possible for any reason.
- Unable to perform a full NIHSS evaluation due to clinical status (coma / intubated).
- Pregnancy.

### Information and consent

Informed written consent is obtained following the completion of emergency treatment aboard the MSU. If patients are unable to provide consent, it is obtained from their legal representative. If immediate consent cannot be obtained during the prehospital emergency intervention, it will be requested later by the study team from the patient or their legal representative.

### Premature withdrawal of a patient from the study

Participation in the study is voluntary. Participants may withdraw from the study at any time, prematurely and without providing a reason, without any impact on their subsequent medical treatment. If a participant declines to participate, any collected samples and associated data will be destroyed. However, once blood samples and data have been anonymized in the regular course of the study, specific destruction or deletion is no longer feasible.

## Endpoints

The LVOCheck-EVA study aims to evaluate an affordable POC test for the early prediction of LVO. The test is intended for prehospital use in standard ambulances to enable accurate prehospital selection and triage of LVO-patients for direct transport to thrombectomy-capable centers, in line with a “targeted mothership” approach. The primary objective of the LVOCheck-Eva subproject is to assess the real-world diagnostic accuracy of the newly developed POCT for estimating LVO probability in patients with suspected acute stroke. The generated data will help to assess the feasibility of integrating this POCT-tool into standard operating procedures for regular ambulances.

### Primary endpoints

The primary endpoint of the study is the accuracy of LVOCheck, integrating patient-specific clinical data and POCT-based measurements of H-FABP, D-Dimer, and NT-proBNP, to predict LVO probability, evaluated against the gold-standard final hospital diagnosis, expressed as sensitivity and specificity.

### Secondary endpoints

The secondary endpoints are divided in three main areas of interest:

First, the study will assess the matrix equivalence between whole blood and plasma samples for NT-proBNP, D-Dimer, and H-FABP measurements using the LVOCheck device.

Secondly, the study will perform a qualitative assessment of the possible future clinical utility of LVOCheck in guiding pre-hospital triage decisions through physician-reported evaluations regarding the impact of individual biomarker concentrations and the overall risk score on clinical decision-making and patient transport strategies.

Finally, the study will evaluate the integration of LVOCheck into clinical workflows through structured collection and analysis of HCPs feedback on practical implementation and compatibility with routine care protocols.

### Safety endpoints

Technical performance evaluated by the frequency and severity of test malfunctions reported through the Mobile App, as well as the rate of critical LVOCheck failures.

The proportion of false-positive assessments (non-LVO patients classified as high-probability LVO) and false-negative assessments (LVO patients not classified as high-probability LVO) by this technology.

## Collection and processing of data and samples

**Figure 1:**
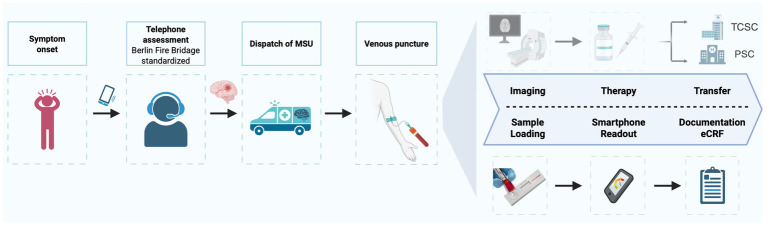
Overview of the clinical (top) and parallel study-related (down) workflows. TCSC, Thrombectomy Capable Stroke Center; PSC, Primary Stroke Center.

Patients with symptoms of acute stroke receive intravenous access for drug administration, contrast agent application and blood sample collection as part of the clinical routine. Directly after venipuncture, extra samples will be obtained without requiring an additional venipuncture. A small volume (26.9 μL) of whole blood will be dispensed with a U-Pette (LabCon, 3,700 Lakeville Highway, Petaluma, California USA), directly adjusted to the heparin vacutainer, adding the buffer just after the blood has been completely absorbed for immediate POC testing. After a 10-min incubation, the LFA results are scanned using the Mobile App. The resulting biomarker concentrations from the test are combined with selected clinical parameters (e.g., age, blood pressure, stroke score) for automated processing, and output of the predictive risk of LVO. The cloud component runs on the established and secure Extra Horizon platform^1^, adhering to ISO 27001:2017 and ISO 27701:2019 standards. The results displayed by the Mobile App are directly saved in the communication system WebPortal and additional clinical data are manually entered into the electronic case report form (eCRF) in Research electronic data capture (REDCap) by the treating MSU physician (Figure 1).

The collection tubes are pre-packed and labeled with unique alphanumeric codes generated by the Charité independent data trust office. These codes include the prefix “LVO- “and a 9-digit random number.

For blood collection, 3 EDTA- tube (4 mL, purple lid) and one Heparin-tube (4 mL, green lid; BD Vacutainer®, Becton, Dickinson and Company, Franklin Lakes, New Jersey, United States) are utilized. Following collection, the tubes are inverted 8–10 times to mix the blood with the preservant and processed at room temperature within 30 min.

The samples are then centrifuged at 2,000 g for 15 min (CompactStar CS4, by avantor™, delivered by vwr™, Radnor, PA, USA, and CD-0506, Phoenix Instrument, Garbsen, Germany). Additionally, a second POCT will be conducted using 15 μL of heparin plasma obtained post-centrifugation, and results will be documented as described above. Finally, the remaining volume of plasma samples is pipetted and divided into 250 μL aliquots in cryotubes, maintaining the color code for each sample type (e.g., Plasma EDTA in purple-lid cryotubes and Heparin in green-lid cryotubes). These cryotubes are placed in a storage box with individual compartments for each sample.

The storage box is placed in a styrofoam container and kept in the MSU refrigerator at 4 °C before being transferred to a − 20 °C freezer at the MSU site within 30 min to 4 h. Within four weeks, samples are transported to the biobank at Charité—Campus Benjamin Franklin, where they are stored at −80 °C. Sample and box positions are recorded throughout the process (Supplementary Figure 1). Patients will have the option to donate the remaining volume of blood samples for additional research purposes, such as the evaluation of other biomarkers. Residual plasma samples will be processed and stored as described above.

Study-related use of analysis results and/or additional blood samples will begin only after written informed consent is obtained from the patient or their legal representative. If written consent is not provided within 12 weeks of sample collection, the patient is classified as a dropout, and all associated samples and data are destroyed.

### Description of the LVOCheck POCT device

In the initial clinical study subproject, LVOCheck-Opti (DRKS00030399), plasma samples were collected that allowed the technical performance (analytical accuracy) of the LFA. LVOCheck is a device under development by the project partner Advanced Brain Companion Diagnostics, S. L. (ABCDx, 167 C/ Sant Antoni Maria Claret, 08025 Barcelona, Spain). This test is currently under development and not available for clinical use.

This portable, non-invasive and rapid solution is designed to predict LVO risk in pre-hospital emergency settings by integrating a blood-based multi-biomarker single LFA– measuring NT-proBNP, D-Dimer and H-FABP– with patient-specific clinical parameters all accessible and guided through a Mobile App.

The single LFA within the assay consists of a sample application pad, a conjugate pad preloaded with analyte-specific antibodies conjugated to a detection label, and a nitrocellulose membrane containing immobilized capture antibodies specific to either NT-proBNP, D-Dimer or H-FABP. Upon blood sample application, a buffer solution must be added to facilitate sample migration through the control and three biomarker test lines on the LFA strip.

After the incubation period of 10 min, results can be easily scan using the App, just positioning the smartphone above the POCT. The scan measures the intensity of the control and test lines to generate numerical signals based on colorimetry for each biomarker, which are ultimately converted into biomarker concentrations using lot-specific calibration curves.

The LFA is designed for use with a digital reader that quantifies the intensity of the test line signals and integrates clinical parameters for enhanced diagnostic accuracy. Respective thresholds have been determined with samples and clinical data from the first subproject, LVOCheck-Opti, as well as the BIO-FAST study (NCT04612218).

Additionally, patient-specific clinical data is registered by the HCP through the App, including patient ID number, age, blood pressure and neurological scale assessment [to choose between: NIHSS (National Institutes of Health Stroke Scale), Cincinnati (Cincinnati Prehospital Stroke Severity Scale), RACECAT (Rapid Arterial Occlusion Evaluation for CATalonia), G-FAST (Cincinnati Stroke Scale-Modified for Gaze, Face, Arm, Speech, and Tim)]. No data allowing for patient identification is entered into the Mobile App. For study purposes, full NIHSS assessment will be performed through the Mobile App.

Finally, after integrating the biomarker concentrations and patient-specific clinical parameters, the predictive LVO risk is calculated by the software component. The Mobile App temporarily displays output data (biomarker concentrations and LVO stroke risk/probability) alongside the clinical input data (age, blood pressure, stroke assessment score), while simultaneously being transmitted to the WebPortal communication system for secure and long-term storage. Additional clinical information, comprising patient final diagnosis, is documented by the treating physician in the eCRF. Due to the MSU’s specialized staffing structure, brief sample handling, and automated smartphone-based notifications, the proposed POC test integrates seamlessly into routine clinical workflows, allowing patient care to proceed without interruption and without prolonging on-scene time.

### Data storage and management

All clinical data collected and biosamples from participants are pseudonymized by assigning a unique identification number, generated by the Charité data trust office (CHA-THS). Each study site maintains a secure pseudonymization list linking identification numbers to participants’ full names and dates of birth for potential identification if necessary. This list remains confidential, is stored exclusively at the study site, and will be archived for at least 10 years post-study completion. Pseudonymized data is entered into an eCRF and managed using REDCap, hosted by Charité—Universitätsmedizin Berlin. Additionally, study participation is documented in the patient’s medical record.

The Mobile App securely manages patient session data for up to 24 h. During this period, users can access patient information—including input data, vascular biomarker concentrations (NT-proBNP, D-Dimer and H-FABP), and calculated LVO stroke probability—by entering their personal PIN code. After 24 h, all data is automatically deleted from the Mobile App.

For long-term storage, patient sessions are transferred to a secure cloud infrastructure and can be viewed through the LVOCheck communication system Access to this portal is restricted to ABCDx administrators only. All entries are organized and sortable by project ID and participant code.

## Definitions

This study will adhere to the definitions previously outlined in the LVOCheck-Opti subproject.

### Ischemic stroke

An episode of neurological dysfunction caused by focal cerebral, spinal, or retinal infarction, while infarction is brain, spinal cord, or retinal cell death attributable to ischemia, based on pathological imaging or clinical evidence of cerebral, spinal cord, or retinal focal ischemic injury based on symptoms persisting ≥24 h or until death, and other etiologies excluded.

### Large-vessel occlusion

Occlusion of blood vessels supplying the brain, detectable by angiography, generally comprising the Internal Carotid Artery (ICA), Middle Cerebral arteries segments 1 and 2 (M1 and M2) and Basilar Artery (BA).

### Intracerebral hemorrhage

Rapidly developing clinical signs of neurological dysfunction attributable to a focal collection of blood within the brain parenchyma or ventricular system that is not caused by trauma.

### Stroke mimic

Certain diseases like syncope, epileptic seizure, hypoglycemia, hyperglycemia, migraine aura, dissociative disorder etc. that can cause similar symptoms as stroke. A stroke is initially clinically suspected, but the diagnostic criteria of stroke are not fulfilled.

## Statistics

### Sample size estimations

The sample size was determined based on the precision achievable for sensitivity and specificity estimates, as measured by confidence interval (CI) widths. We estimate that 14.4% of stroke-alert patients will have an LVO diagnosis, based on prior data (internal STEMO data) derived from a preliminary study where 260 of 1,800 patients were diagnosed with LVO. We anticipate achieving a sensitivity of at least 75% and a specificity of at least 95%. For a cohort of 800 patients, the 95% CI for sensitivity would span ±8.26 percentage points (75% ± 8.26%), while the 95% CI for specificity would span ±1.7 percentage points (95% ± 1.7%). These levels of precision are considered sufficient for determining the diagnostic accuracy of the LVOCheck multiplex tool in this new cohort.

## Statistical analysis

A detailed statistical analysis plan will be provided before the analyses of the data. Descriptive statistics, including mean, standard deviation, median, and interquartile range (IQR), will be reported for key variables such as patient age. Predictions for each patient will be generated using the regression model developed in the previous phase and compared against the confirmed diagnoses to estimate sensitivity, specificity, accuracy, and corresponding 95% CIs. Exploratory subgroup analyses will be conducted to explore potential differences across demographic or clinical strata.

### Statistical analysis of the primary hypothesis

The definitive diagnosis of LVO as documented in the physician’s letter will serve as the reference standard for the analysis (Comparator). Multiple predictive models will be constructed to estimate each patient’s probability of LVO. Independent variables will include H-FABP, D-Dimer and NT-proBNP, and relevant clinical parameters readily available in the acute setting. The primary goal is to assess whether incorporating biomarker levels measured through POCT along with an LVO prediction diagnostic accuracy beyond what can be achieved with standard clinical information alone. Consequently, models that include both biomarkers and baseline clinical data will be compared against models featuring only clinical data. Key metrics such as sensitivity, specificity, positive predictive value (PPV) and negative predictive values (NPV) will be calculated to assess the diagnostic accuracy in detecting LVO in suspected stroke patients. Receiver Operating Characteristic (ROC) analysis will be used to evaluate overall diagnostic performance, with confidence intervals computed for all metrics to assess precision. Various predictive models will be examined iteratively which sensitivity is maximized while preserving a specificity level of at least 92.5%. This approach will characterize the real-world diagnostic accuracy of the POCT for LVO-prediction.

### Statistical analysis of the secondary hypothesis

The statistical analysis for the secondary endpoints will focus on evaluating the impact of the newly developed tool on triage decisions, and clinical workflow integration. First, the matrix equivalence between blood and plasma measurements will be assessed using Bland–Altman plots and calculating correlation coefficients to determine agreement and potential systematic biases.

For the second endpoint, the effectiveness in guiding triage decisions will be analyzed using descriptive statistics and cross-tabulation to assess any patterns in how biomarker information and the predictive LVO probability correlates with triage decisions, assessed by simple questions included in the eCRF.

Finally, for evaluating its integration into clinical workflow, qualitative feedback from HCPs will be collected and analyzed using thematic analysis or Likert scale responses to assess the LVOCheck tool’s practicality in routine care. Descriptive statistics and possibly chi-square tests will be used to assess HCP satisfaction and feasibility of incorporating this multiplex tool into clinical protocols.

## Discussion

This real-world study aims to evaluate the diagnostic accuracy and feasibility of the multiplex LVOCheck tool, considering human factors and usability. Specifically, it investigates whether measurement of the biomarkers H-FABP, D-Dimer and NT-proBNP using a LFA as part of a POCT, combined with patient-specific medical information and supported by a Mobile App, can enhance the detection of LVO in patients with suspected acute stroke in a prehospital ambulance setting by predicting the risk of LVO stroke. To our knowledge, this is the first large-scale clinical study to investigate the combined measurement of these biomarkers at the POC, integrated with a smartphone application.

Several prior studies investigated the diagnostic utility of NT-proBNP and H-FABP, as well as D-Dimer in different scenarios.

Bustamante et al. (18) analyzed NT-proBNP levels alongside other biomarkers within the STROKE-CHIP study in 1,308 patients with suspected stroke presenting within 6 hours of symptom onset. Their findings demonstrated that combining elevated NT-proBNP and endostatin levels with clinical parameters a predictive accuracy of 80.6% for distinguishing ischemic from hemorrhagic strokes can be achieved.

Previous smaller case–control studies have reported significantly higher NT-proBNP levels in patients with AIS compared to healthy controls. AIS patients with infarct diameters exceeding 3 cm exhibited higher NT-proBNP concentrations compared to controls (log NT-proBNP: 7.96 ng/mL (SD 1.66) vs. 6.52 ng/mL (SD 1.6); *p* = 0.002). Notably, NT-proBNP levels were significantly elevated in AIS patients with electrocardiographic changes, particularly among those with reduced left ventricular ejection fraction or impaired left ventricular end-diastolic diameter (*p* = 0.019 and *p* = 0.011) (19). No significant differences in NT-proBNP levels were observed based on whether the stroke occurred in the anterior or posterior vascular territories (20).

Park et al. observed elevated H-FABP levels in acute ischemic stroke (AIS) patients compared to healthy controls in a case–control study involving 111 AIS patients and 127 healthy controls. Similarly, Zimmermann-Ivol et al. reported elevated H-FABP levels in AIS patients (*n* = 22) compared to healthy controls (*n* = 22) in a small case–control study, achieving a sensitivity of 68.2% and a specificity of 100% (28).

Guaman-Pilco et al. recently conducted a retrospective analysis of H-FABP levels in 175 blood samples from the STROKE-CHIP study using a POC testing tool. Their results demonstrated significantly higher H-FABP levels in individuals with TIA compared to stroke mimics [3.10 ng/mL (IQR 2.13–4.78) vs. 1.70 ng/mL (IQR 1.23–2.38); *p* < 0.001], suggesting that H-FABP may have potential as a diagnostic marker for TIA (29).

Elevated D-Dimer levels have been associated with an increased risk of recurrent stroke and poorer short-term outcomes in AIS patients. Its diagnostic utility in acute stroke diagnosis has primarily been explored in studies evaluating biomarker panels that combine multiple markers to distinguish AIS mainly from healthy controls or stroke mimics (28, 30–33); but it has also been reported as a predictor of LVO cases (34). However, most of these studies collected blood samples at relatively late time points of up to 24 h after symptom onset and none demonstrated diagnostic performance appropriate for clinical application.

In a more recent trial, Durrani et al. investigated the diagnostic accuracy of the measurement of GFAP (cutoff concentration 213 pg./mL) and D-Dimer (600 ng/mL) in combination with the FAST-ED score specifically for LVO detection in patients with suspected stroke within 18 h after symptom onset. They observed a diagnostic accuracy for LVO detection with a specificity of 94% and a sensitivity of 71% (35). Interestingly, the performance increased in a subgroup analysis of patients presenting within 6 h after symptom onset (specificity 93%, sensitivity 81%).

Recently, Kowalski et al. conducted a small prospective case–control study involving blood sample collection aboard a mobile stroke unit, with a median onset-to-sampling time of 2.22 h (36). The study demonstrated a significant increase in inflammatory and neurotoxicity markers in the serum and extracellular vesicles of AIS patients compared to healthy controls already as early as 36 min after symptom onset. These findings highlight the importance and necessity of studying stroke biomarkers in the ultra-early phase after symptom onset, preferably using mobile stroke units with advanced pre-analytical capabilities.

Taken together LVOCheck-Eva study addresses a common limitation of stroke biomarker research by conducting POCT measurements and blood sampling directly on board the MSU: late and variable sampling conditions. In this study, blood is collected within minutes of patient contact, allowing technicians to perform POCT measurements and sample processing immediately. This approach minimizes delays while ensuring optimal pre-analytical quality and represents the intended use case of prehospital biomarker measurement.

Although the study design allows enrolling patients up to 24 h after symptom onset, previous studies conducted aboard the Berlin MSU network showed that most patients will be assessed very early (5, 11), providing an opportunity to characterize biomarker levels during a critically more relevant period with respect to treatment and transportation decisions.

LVOCheck-Eva uses the MSU environment to integrate POCT testing to predict LVO risk directly into prehospital workflows. By using a dedicated POCT tool, biomarkers measurements are conducted on-site and in real-time, potentially enabling faster decision-making while closely reflecting the intended future use case of point of care testing in standard ambulances.

Due to – in most cases – very early patient contact after symptom onset, this approach allows to understand how these biomarkers behave shortly after onset and to test whether early dynamics can be reliably measured using a LFA-based POCT architecture. To the best of our knowledge, LVOCheck-EVA is the largest clinical study focused on integrating fully prehospital POCT-based biomarker measurements with software to predict LVO risk in stroke care. The scale and setting will allow us to assess not only the diagnostic accuracy of H-FABP, D-Dimer and NT-proBNP together with the LVO prediction but also to gather information about the logistical and operational feasibility of deploying LVOCheck in the prehospital setting.

The current study focuses on LVO prediction using biomarkers previously associated with ischemic stroke subtypes. NT-proBNP, D-Dimer, and H-FABP were selected for their relevance to cardioembolic sources, thromboembolic activity, and neuronal or myocardial injury, respectively – mechanisms predominantly linked to ischemic rather than hemorrhagic events. Accordingly, the panel has limited ability to differentiate ischemia from intracerebral hemorrhage. As reliably distinguishing ICH from ischemic stroke remains a major prehospital challenge, future research could explore adding complementary biomarkers with strong diagnostic accuracy, such as GFAP or UCH-L1 for ICH detection (37–39), potentially improving the overall diagnostic accuracy of prehospital LFA-based POCT for distinguishing between AIS, ICH, and stroke mimics.

## Conclusion

This study aims to evaluate the multiplex POCT in real-world environments, specifically within the Berlin MSU network, which participated in the previous LVOCheck-Opti clinical study. The novel tool quantifies H-FABP, D-Dimer and NT-proBNP using a single multiplex LFA and a Mobile App, to predict LVO in the ultra-early prehospital phase of AIS at the POC, such as in ambulances. Integrating such biomarker and LVO predictive risk-based diagnostics into prehospital care could support early decision-making and triage for suspected LVO stroke patients, potentially reducing unnecessary transfers and time to critical interventions such as thrombectomy, thereby improving patient outcomes. Currently, no biomarkers have shown adequate diagnostic accuracy to reliably guide these critical decisions in the ultra-early phase after symptom onset.

## Footnotes

1
www.extrahorizon.com/solutions/platform-regulations

## Glossary

AIS: Acute ischemic stroke
ATT: Alarm-to-treatment time
AUC: Area under the curve
BMBF: Bundesministerium für Bildung und Forschung
CHA-THS: Charité data trust office
Cincinnati (CPSSS): Cincinnati Prehospital Stroke Severity Scale
CSB: Centrum für Schlaganfallforschung Berlin
CT: Computed tomography
CCT: Cranial computed tomography
CTA: Computed tomography angiography
DD: Differential diagnosis
DLR: Deutsches Zentrum für Luft-und Raumfahrt
eCRF: Electronic case report form
EDTA: Ethylenediaminetetraacetic acid
ELISA: Enzyme-linked immunosorbent assay
ESOC: European Stroke Organization Conference
(H-)FABP: h-Fatty acid binding protein
IVD: *in vitro* diagnostic
GCP: Good clinical practice
GFAP: Glial fibrillary acid protein
G-FAST: Cincinnati Stroke Scale-Modified for Gaze, Face, Arm, Speech, and Tim
ICH: Intracerebral Hemorrhage
INR: International normalized ratio
JAMA: Journal of the American Medical Association
LFA: Lateral Flow Assay
LVO: Large vessel occlusion
MD: Medical Device
ML: Machine Learning
mRS: Modified Rankin scale
MRI: Magnetic resonance imaging
MSU: Mobile stroke unit
MT: Mechanical thrombectomy
NIHSS: National Institutes of Health Stroke Scale
NT-proBNP: N-terminal prohormone of brain natriuretic peptide
PI: Principal investigator
POC: Point-of-care
POCT: POC test
PRESTO: Prehospital Stroke Organization
PTT: Partial thromboplastin time
RACECAT: Rapid Arterial Occlusion Evaluation for CATalonia
REDCAP: Research electronic data capture
ROC: Receiver operating characteristic
SM: Stroke Mimic
STEMO: Stroke Einsatz Mobil
TIA: Transient ischemic attack
TT: Thrombin time

## References

[1] Steinmetz JD Seeher KM Schiess N Nichols E Cao B Servili C . Global, regional, and national burden of disorders affecting the nervous system, 1990–2021: a systematic analysis for the global burden of disease study 2021. Lancet Neurol. (2024) 18:344–81. doi: 10.1016/S1474-4422(24)00038-3 PMC10949203 38493795

[2] Berge E Whiteley W Audebert H De Marchis G Fonseca AC Padiglioni C . European stroke organisation (ESO) guidelines on intravenous thrombolysis for acute ischaemic stroke. Eur Stroke J. (2021) 6:1–63. doi: 10.1177/2396987321989865, 33817340 PMC7995316

[3] Turc G Bhogal P Fischer U Khatri P Lobotesis K Mazighi M . European stroke organisation (ESO) – European Society for Minimally Invasive Neurological Therapy (ESMINT) guidelines on mechanical Thrombectomy in acute Ischaemic StrokeEndorsed by stroke Alliance for Europe (SAFE). Eur Stroke J. (2019) 4:6–12. doi: 10.1177/2396987319832140, 31165090 PMC6533858

[4] Turc G Tsivgoulis G Audebert HJ Boogaarts H Bhogal P De Marchis GM . European stroke organisation – European Society for Minimally Invasive Neurological Therapy expedited recommendation on indication for intravenous thrombolysis before mechanical thrombectomy in patients with acute ischaemic stroke and anterior circulation large vessel occlusion. Eur Stroke J. (2022) 7:1–26. doi: 10.1177/23969873221076968 PMC8921785 35300256

[5] Ebinger M Kunz A Wendt M Rozanski M Winter B Waldschmidt C . Effects of golden hour thrombolysis: a prehospital acute neurological treatment and optimization of medical Care in Stroke (PHANTOM-S) substudy. JAMA Neurol. (2015) 72:25–30. doi: 10.1001/jamaneurol.2014.3188, 25402214

[6] Al-Ajlan FS Alkhiri A Alamri AF Alghamdi BA Almaghrabi AA Alharbi AR . Golden hour intravenous thrombolysis for acute ischemic stroke: a systematic review and Meta-analysis. Ann Neurol. (2024) 96:582–90. doi: 10.1002/ana.27007, 38922985

[7] Navi BB Bach I Czap AL Wang M Yamal JM Jacob AP . Strokes averted by intravenous thrombolysis: a secondary analysis of a prospective, multicenter, controlled trial of Mobile stroke units. Ann Neurol. (2024) 95:347–61. doi: 10.1002/ana.26816, 37801480

[8] Goyal M Ospel JM Ganesh A Dowlatshahi D Volders D Möhlenbruch MA . Endovascular treatment of stroke due to medium-vessel occlusion. N Engl J Med. (2025) 392:1385–95. doi: 10.1056/NEJMoa2411668 39908448

[9] Psychogios M Brehm A Ribo M Rizzo F Strbian D Räty S . Endovascular treatment for stroke due to occlusion of medium or distal vessels. N Engl J Med. (2025) 392:1374–84. doi: 10.1056/NEJMoa2408954, 39908430

[10] Grotta JC Yamal JM Parker SA Rajan SS Gonzales NR Jones WJ . Prospective, multicenter, controlled trial of Mobile stroke units. N Engl J Med. (2021) 385:971–81. doi: 10.1056/NEJMoa2103879, 34496173

[11] Ebinger M Siegerink B Kunz A Wendt M Weber JE Schwabauer E . Association between dispatch of Mobile stroke units and functional outcomes among patients with acute ischemic stroke in Berlin. JAMA. (2021) 325:454–66. doi: 10.1001/jama.2020.26345, 33528537 PMC7856548

[12] Turc G Hadziahmetovic M Walter S Churilov L Larsen K Grotta JC . Comparison of Mobile stroke unit with usual Care for Acute Ischemic Stroke Management: a systematic review and Meta-analysis. JAMA Neurol. (2022) 79:281–90. doi: 10.1001/jamaneurol.2021.5321, 35129584 PMC8822443

[13] Ebinger M Rozanski M Waldschmidt C Weber J Wendt M Winter B . PHANTOM-S: the prehospital acute neurological therapy and optimization of medical care in stroke patients - study. Int J Stroke. (2012) 7:348–53. doi: 10.1111/j.1747-4949.2011.00756.x, 22300008

[14] Pérez de la Ossa N Abilleira S Jovin TG García-Tornel Á Jimenez X Urra X . Effect of direct transportation to Thrombectomy-capable center vs local stroke center on neurological outcomes in patients with suspected large-vessel occlusion stroke in nonurban areas: the RACECAT randomized clinical trial. JAMA. (2022) 327:1782–94. doi: 10.1001/jama.2022.4404, 35510397 PMC9073661

[15] Helwig SA Ragoschke-Schumm A Schwindling L Kettner M Roumia S Kulikovski J . Prehospital stroke management optimized by use of clinical scoring vs Mobile stroke unit for triage of patients with stroke: a randomized clinical trial. JAMA Neurol. (2019) 76:1484–92. doi: 10.1001/jamaneurol.2019.2829, 31479116 PMC6724153

[16] Oliveira Gonçalves AS Rohmann JL Piccininni M Kurth T Ebinger M Endres M . Economic evaluation of a Mobile stroke unit Service in Germany. Ann Neurol. (2023) 93:942–51. doi: 10.1002/ana.26602, 36637359

[17] Rink JS Szabo K Hoyer C Saver JL Nour M Audebert HJ . Mobile stroke units services in Germany: a cost-effectiveness modeling perspective on catchment zones, operating modes, and staffing. Eur J Neurol. (2025) 32:e16514. doi: 10.1111/ene.16514, 39506352 PMC11622509

[18] Bustamante A López-Cancio E Pich S Penalba A Giralt D García-Berrocoso T . Blood biomarkers for the early diagnosis of stroke: the stroke-Chip study. Stroke. (2017) 48:2419–25. doi: 10.1161/STROKEAHA.117.017076, 28716979

[19] Iltumur K Yavavli A Apak I Ariturk Z Toprak N. Elevated plasma N-terminal pro-brain natriuretic peptide levels in acute ischemic stroke. Am Heart J. (2006) 151:1115–22. doi: 10.1016/j.ahj.2005.05.022, 16644347

[20] Giannakoulas G Hatzitolios A Karvounis H Koliakos G Charitandi A Dimitroulas T . N-terminal pro-brain natriuretic peptide levels are elevated in patients with acute ischemic stroke. Angiology. (2005) 56:723–30. doi: 10.1177/000331970505600610, 16327949

[21] Ageno W Finazzi S Steidl L Biotti MG Mera V Melzi d’Eril G . Plasma measurement of D-dimer levels for the early diagnosis of ischemic stroke subtypes. Arch Intern Med. (2002) 162:2589–93. doi: 10.1001/archinte.162.22.2589 12456231

[22] Zhang P Wang C Wu J Zhang S. A systematic review of the predictive value of plasma D-dimer levels for predicting stroke outcome. Front Neurol. (2021) 12:693524. doi: 10.3389/fneur.2021.693524 34295302 PMC8289899

[23] Zimmermann-Ivol CG Burkhard PR Le Floch-Rohr J Allard L Hochstrasser DF Sanchez JC. Fatty acid binding protein as a serum marker for the early diagnosis of stroke: a pilot study. Mol Cell Proteomics. (2004) 3:66–72. doi: 10.1074/mcp.M300066-MCP200, 14581522

[24] Dagonnier M Donnan GA Davis SM Dewey HM Howells DW. Acute stroke biomarkers: are we there yet? Front Neurol. (2021) 12:619721. doi: 10.3389/fneur.2021.619721 33633673 PMC7902038

[25] Menéndez-Valladares P Delgado M Núñez-Jurado D Sempere-Bordes L Penalba A Azurmendi L . Smartphone-enabled point-of-care testing for prehospital stroke diagnosis. Prehosp Emerg Care. (2024) 12:1–10. doi: 10.1080/10903127.2024.2437657, 39630146

[26] Villalonga JM. (2021). Biomarkers for initiating onsite and faster ambulance stroke therapies (BIO-FAST). Available online at: https://clinicaltrials.gov/study/NCT04612218. [Accessed December 20, 2024]

[27] Kaffes M Bondi F Geisler F Grittner U Haacke L Ihl T . Optimization of sensitivity and specificity of a biomarker-based blood test (LVOCheck-Opti): a protocol for a multicenter prospective observational study of patients suspected of having a stroke. Front Neurol. (2023) 14:1327348. doi: 10.3389/fneur.2023.1327348, 38371304 PMC10870936

[28] Park SY Kim MH Kim OJ Ahn HJ Song JY Jeong JY . Plasma heart-type fatty acid binding protein level in acute ischemic stroke: comparative analysis with plasma S100B level for diagnosis of stroke and prediction of long-term clinical outcome. Clin Neurol Neurosurg. (2013) 115:405–10. doi: 10.1016/j.clineuro.2012.06.004, 22766253

[29] Guamán-Pilco D Chocano E Palà E Lamana-Vallverdú M Penalba A García-Rodríguez P . H-FABP as a biomarker in transient ischemic attack. J Cardiovasc Transl Res. (2024) 18:40–7. doi: 10.1007/s12265-024-10552-4, 39160445

[30] Kim MH Kang SY Kim MC Lee WI. Plasma biomarkers in the diagnosis of acute ischemic stroke. Ann Clin Lab Sci. (2010) 40:336–41. 20947807

[31] Montaner J Mendioroz M Ribó M Delgado P Quintana M Penalba A . A panel of biomarkers including caspase-3 and D-dimer may differentiate acute stroke from stroke-mimicking conditions in the emergency department. J Intern Med. (2011) 270:166–74. doi: 10.1111/j.1365-2796.2010.02329.x, 21198992

[32] Reynolds MA Kirchick HJ Dahlen JR Anderberg JM McPherson PH Nakamura KK . Early biomarkers of stroke. Clin Chem. (2003) 49:1733–9. 14500614 10.1373/49.10.1733

[33] Laskowitz DT Kasner SE Saver J Remmel KS Jauch EC BRAIN Study Group. Clinical usefulness of a biomarker-based diagnostic test for acute stroke: the biomarker rapid assessment in ischemic injury (BRAIN) study. Stroke. (2009) 40:77–85. doi: 10.1161/STROKEAHA.108.516377, 18948614

[34] Ramos-Pachón A López-Cancio E Bustamante A Pérez de la Ossa N Millán M Hernández-Pérez M . D-dimer as predictor of large vessel occlusion in acute ischemic stroke. Stroke. (2021) 52:852–8. doi: 10.1161/STROKEAHA.120.031657, 33563016

[35] Durrani Y Gerstl JVE Murphy D Harris A Saali I Gropen T . Prospective validation of glial fibrillary acidic protein, d-dimer, and clinical scales for acute large-vessel occlusion ischemic stroke detection. Stroke Vasc Interv Neurol. (2024) 4:e001304. doi: 10.1161/SVIN.123.001304 PMC12778543 41585381

[36] Kowalski RG Ledreux A Violette JE Neumann RT Ornelas D Yu X . Rapid activation of Neuroinflammation in stroke: plasma and extracellular vesicles obtained on a Mobile stroke unit. Stroke. (2023) 54:e52–7. doi: 10.1161/STROKEAHA.122.041422, 36727508 PMC10052772

[37] Luger S Jæger HS Dixon J Bohmann FO Schaefer J Richieri SP . Diagnostic accuracy of glial fibrillary acidic protein and ubiquitin carboxy-terminal hydrolase-L1 serum concentrations for differentiating acute intracerebral hemorrhage from ischemic stroke. Neurocrit Care. (2020) 33:39–48. doi: 10.1007/s12028-020-00931-5, 32096121

[38] Jæger HS Tranberg D Larsen K Valentin JB Blauenfeldt RA Luger S . Diagnostic performance of glial fibrillary acidic protein and prehospital stroke scale for identification of stroke and stroke subtypes in an unselected patient cohort with symptom onset < 4.5 h. Scand J Trauma Resusc Emerg Med. (2023) 31:1. doi: 10.1186/s13049-022-01065-7, 36604741 PMC9814331

[39] Rozanski M Waldschmidt C Kunz A Grittner U Ebinger M Wendt M . Glial fibrillary acidic protein for prehospital diagnosis of intracerebral hemorrhage. Cerebrovasc Dis. (2017) 43:76–81. doi: 10.1159/000453460, 27951536

