# Supplementary figures and images for: Evaluation of the prehospital diagnostic accuracy of a novel point-of-care test for NT-proBNP, D-dimer and H-FABP and large-vessel occlusion risk assessment (LVOCheck-EVA): a protocol for a multicenter prospective observational study in patients suspected of having a stroke
Source: Front Neurol. 2025 Dec 8;16:1697711. doi: 10.3389/fneur.2025.1697711 (PMC12719076; doi:10.3389/fneur.2025.1697711)

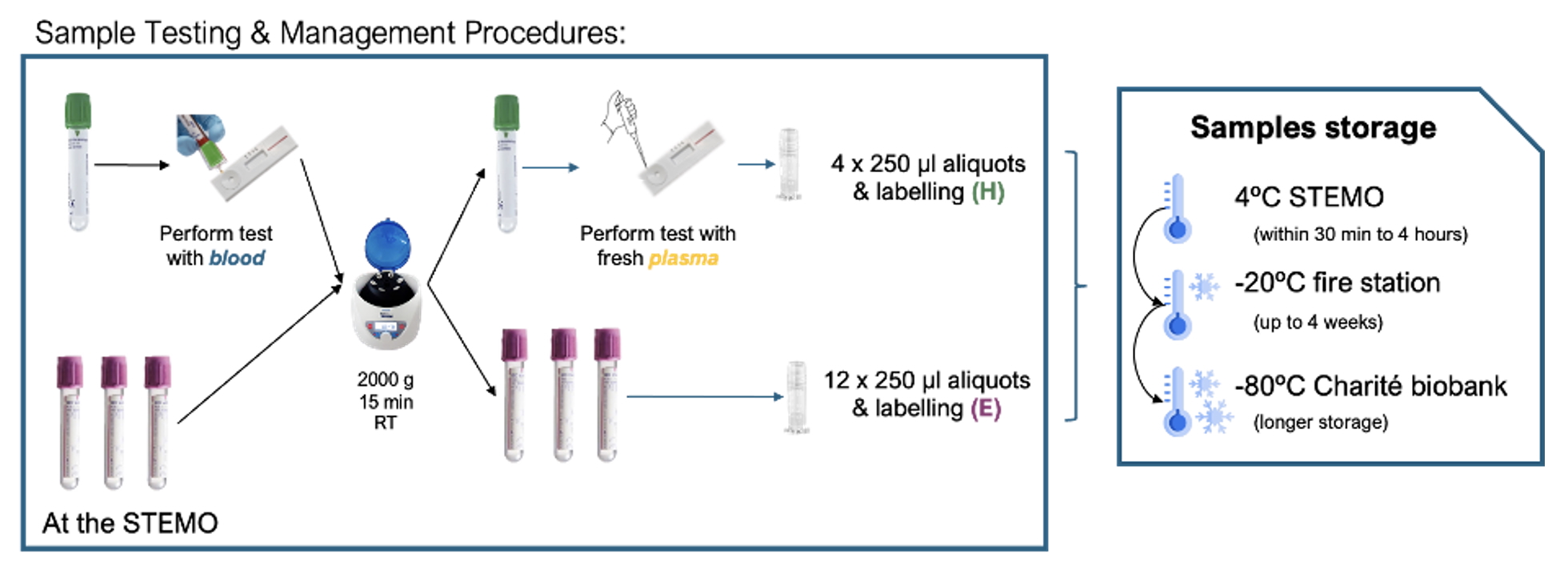

Supplement: Supplementary file 1 [file Image_1.JPEG]
